# Supplementary material for: Pollinator diversity and reproductive success of Epipactis helleborine (L.) Crantz (Orchidaceae) in anthropogenic and natural habitats
Source: PeerJ. 2017 Apr 18;5:e3159. doi: 10.7717/peerj.3159 (PMC5398293; doi:10.7717/peerj.3159)
Supplement: Table S1 — (A) In anthropogenic habitats (A1–A4), (B) Vascular flora in natural habitats (N1–N4). [file peerj-05-3159-s001.docx]

**Supplementary Table 1. Raw data. A) Vascular flora in anthropogenic habitats (A1-A4), B) Vascular flora in natural habitats (N1-N4)**

A)

| No. | Species | A1 | A2 | A3 | A4 | Fi (%) |
| --- | --- | --- | --- | --- | --- | --- |
| 1. | *Achillea millefolium* | + | + | + | + | 100 |
| 2. | *Conyza canadensis* | + | + | + | + | 100 |
| 3. | *Dactylis glomerata* | + | + | + | + | 100 |
| 4. | *Epipactis helleborine* | + | + | + | + | 100 |
| 5. | *Galium aparine* | + | + | + | + | 100 |
| 6. | *Medicago lupulina* | + | + | + | + | 100 |
| 7. | *Plantago media* | + | + | + | + | 100 |
| 8. | *Poa annua* | + | + | + | + | 100 |
| 9. | *Poa nemoralis* | + | + | + | + | 100 |
| 10. | *Vicia cracca* | + | + | + |  | 100 |
| 11. | *Daucus carota* | + | + |  | + | 75 |
| 12. | *Linaria vulgaris* | + | + | + |  | 75 |
| 13. | *Poa ovata* | + |  | + | + | 75 |
| 14. | *Potentilla anserina* | + |  | + | + | 75 |
| 15. | *Ranunculus acris* | + |  | + | + | 75 |
| 16. | *Rumex acetosella* | + | + | + |  | 75 |
| 17. | *Tanacetum vulgare* | + | + |  | + | 75 |
| 18. | *Taraxacum officinale* | + |  | + | + | 75 |
| 19. | *Acer platanoides* |  |  | + | + | 50 |
| 20. | *Melandrium album* | + | + |  |  | 50 |
| 21. | *Mycelis muralis* |  | + | + |  | 50 |
| 22. | *Phleum pratense* | + | + |  |  | 50 |
| 23. | *Picea abies* |  |  | + | + | 50 |
| 24. | *Poa pratensis* |  |  | + | + | 50 |
| 25. | *Polypodium vulgare* |  | + | + |  | 50 |
| 26. | *Populus* sp. |  | + | + |  | 50 |
| 27. | *Robinia pseudoacacia* |  |  | + | + | 50 |
| 28. | *Senecio jacobaea* |  |  | + | + | 50 |
| 29. | *Urtica dioica* | + | + |  |  | 50 |
| 30. | *Artemisia campestris* |  | + |  |  | 25 |
| 31. | *Cirsium vulgare* | + |  |  |  | 25 |
| 32. | *Galium mollugo* | + |  |  |  | 25 |
| 33. | *Oenothera rubricaulis* | + |  |  |  | 25 |
| 34. | *Pinus sylvestris* |  |  |  | + | 25 |
| 35. | *Quercus robur* | + |  |  |  | 25 |
| 36. | *Sambucus nigra* |  |  | + |  | 25 |
| 37. | *Solidago canadensis* | + |  |  |  | 25 |
| 38. | *Veronica chamaedrys* | + |  |  |  | 25 |
| Total number of species | | 16 | 27 | 21 | 25 |  |

B)

| No. | Species | N1 | N2 | N3 | N4 | Fi (%) |
| --- | --- | --- | --- | --- | --- | --- |
| 1. | *Carpinus betulus* | + | + | + | + | 100 |
| 2. | *Epipactis helleborine* | + | + | + | + | 100 |
| 3. | *Rubus sp.* | + | + | + | + | 100 |
| 4. | *Veronica chamaedrys* | + | + | + | + | 100 |
| 5. | *Acer platanoides* | + | + | + |  | 75 |
| 6. | *Aegopodium podagraria* | + |  | + | + | 75 |
| 7. | *Frangula alnus* | + |  | + | + | 75 |
| 8. | *Juniperus communis* | + |  | + | + | 75 |
| 9. | *Picea abies* |  | + | + | + | 75 |
| 10. | *Pinus sylvestris* |  | + | + | + | 75 |
| 11. | *Pteridium aquilinum* |  | + | + | + | 75 |
| 12. | *Quercus robur* | + | + | + |  | 75 |
| 13. | *Convallaria majalis* |  |  | + | + | 50 |
| 14. | *Dactylis glomerata* | + |  | + |  | 50 |
| 15. | *Impatiens parviflora* | + | + |  |  | 50 |
| 16. | *Oxalis acetosella* |  |  | + | + | 50 |
| 17. | *Poa annua* |  |  | + | + | 50 |
| 18. | *Ranunculus acris* |  |  | + | + | 50 |
| 19. | *Sorbus acuparia* | + |  | + |  | 50 |
| 20. | *Stellaria holostea* |  | + |  | + | 50 |
| 21. | *Achillea millefolium* | + |  |  |  | 25 |
| 22. | *Anthriscus sylvestris* |  |  | + |  | 25 |
| 23. | *Corylus avellana* |  |  | + |  | 25 |
| 24. | *Equisetum sylvaticum* |  |  | + |  | 25 |
| 25. | *Fagus sylvatica* |  | + |  |  | 25 |
| 26. | *Fragaria vesca* |  |  | + |  | 25 |
| 27. | *Galium sylvaticum* |  | + |  |  | 25 |
| 28. | *Lysimachia nummularia* |  | + |  |  | 25 |
| 29. | *Maianthemum bifolium* |  |  | + |  | 25 |
| 30. | *Medicago lupulina* |  |  |  | + | 25 |
| 31. | *Melampyrum nemorosum* | + |  |  |  | 25 |
| 32. | *Mycelis muralis* |  |  |  | + | 25 |
| 33. | *Paris quadrifolia* |  |  | + |  | 25 |
| 34. | *Poa pratensis* | + |  |  |  | 25 |
| 35. | *Senecio jacobaea* |  | + |  |  | 25 |
| 36. | *Taraxacum officinale* | + |  |  |  | 25 |
| 37. | *Tilia cordata* |  | + |  |  | 25 |
| 38. | *Vaccinium myrtillus* |  |  | + |  | 25 |
| Total number of species | | 16 | 16 | 25 | 17 |  |
