# Supplementary material for: Pollinator diversity and reproductive success of Epipactis helleborine (L.) Crantz (Orchidaceae) in anthropogenic and natural habitats
Source: PeerJ. 2017 Apr 18;5:e3159. doi: 10.7717/peerj.3159 (PMC5398293; doi:10.7717/peerj.3159)
Supplement: Table S2 — Abbreviations: population code (Pop), height of plants (HP), number of flowers (NF), number of fruit (capsules) (NFR). [file peerj-05-3159-s002.docx]

Supplementary Table 2. Raw data - biometric traits of *E. helleborine* in the studied populations. Abbreviations: population code (Pop), height of plants (HP), number of flowers (NF), number of fruit (capsules) (NFR).

|  | Pop | HP | NF | NFR |  | Pop | HP | NF | NFR |
| --- | --- | --- | --- | --- | --- | --- | --- | --- | --- |
|  | 2011 | | | | | | | | |
| 1 | A1 | 78 | 33 | 35 | 1 | N1 | 82 | 52 | 30 |
| 2 | A1 | 72 | 17 | 16 | 2 | N1 | 75.5 | 14 | 13 |
| 3 | A1 | 116 | 24 | 20 | 3 | N1 | 87 | 17 | 16 |
| 4 | A1 | 119 | 29 | 29 | 4 | N1 | 69 | 13 | 11 |
| 5 | A1 | 105 | 29 | 29 | 5 | N1 | 78 | 35 | 23 |
| 6 | A1 | 111 | 22 | 22 | 6 | N1 | 90 | 35 | 23 |
| 7 | A1 | 120 | 30 | 29 | 7 | N1 | 80 | 29 | 20 |
| 8 | A1 | 121 | 33 | 32 | 8 | N1 | 66 | 14 | 10 |
| 9 | A1 | 107 | 22 | 20 | 9 | N1 | 65.5 | 28 | 14 |
| 10 | A1 | 105 | 23 | 23 | 10 | N1 | 56 | 7 | 13 |
| 11 | A1 | 73 | 14 | 13 | 11 | N1 | 42 | 5 | 0 |
| 12 | A1 | 64 | 15 | 14 | 12 | N1 | 86 | 22 | 15 |
| 13 | A1 | 52 | 10 | 9 | 13 | N1 | 34 | 9 | 0 |
| 14 | A1 | 112 | 36 | 36 | 14 | N1 | 34.5 | 6 | 4 |
| 15 | A1 | 91 | 25 | 24 | 15 | N1 | 36.5 | 8 | 0 |
| 16 | A1 | 93 | 19 | 18 | 16 | N1 | 50 | 14 | 0 |
| 17 | A1 | 92 | 13 | 12 | 17 | N1 | 54 | 25 | 5 |
| 18 | A1 | 48 | 10 | 9 | 18 | N1 | 73 | 23 | 18 |
| 19 | A1 | 42 | 5 | 4 | 19 | N1 | 79 | 24 | 13 |
| 20 | A1 | 76 | 14 | 14 | 20 | N1 | 60 | 12 | 1 |
| 21 | A1 | 56 | 4 | 4 | 21 | N1 | 56 | 14 | 0 |
| 22 | A1 | 103 | 31 | 30 | 22 | N1 | 51 | 8 | 7 |
| 23 | A1 | 71 | 22 | 20 | 23 | N1 | 63 | 21 | 13 |
| 24 | A1 | 106 | 23 | 23 | 24 | N1 | 49 | 12 | 0 |
| 25 | A1 | 76 | 17 | 16 | 25 | N1 | 32 | 6 | 0 |
| 26 | A1 | 89 | 10 | 9 | 26 | N2 | 70 | 42 | 30 |
| 27 | A1 | 58 | 10 | 9 | 27 | N2 | 99 | 32 | 22 |
| 28 | A1 | 79 | 21 | 21 | 28 | N2 | 77 | 23 | 15 |
| 29 | A1 | 107 | 21 | 21 | 29 | N2 | 52 | 16 | 11 |
| 30 | A1 | 52 | 6 | 6 | 30 | N2 | 46 | 13 | 11 |
| 31 | A1 | 101 | 28 | 26 | 31 | N2 | 73 | 24 | 15 |
| 32 | A1 | 98 | 25 | 24 | 32 | N2 | 39.5 | 23 | 15 |
| 33 | A1 | 42 | 2 | 2 | 33 | N2 | 48 | 7 | 4 |
| 34 | A1 | 62 | 13 | 12 | 34 | N2 | 54.2 | 11 | 8 |
| 35 | A1 | 95 | 33 | 32 | 35 | N2 | 56 | 18 | 11 |
| 36 | A1 | 73 | 22 | 21 | 36 | N2 | 37 | 4 | 3 |
| 37 | A1 | 66 | 11 | 6 | 37 | N2 | 48 | 5 | 3 |
| 38 | A1 | 74 | 23 | 10 | 38 | N2 | 45 | 10 | 6 |
| 39 | A1 | 102 | 34 | 33 | 39 | N2 | 35.5 | 12 | 11 |
| 40 | A2 | 83 | 25 | 26 | 40 | N2 | 52 | 10 | 5 |
| 41 | A2 | 67 | 27 | 25 | 41 | N2 | 80 | 34 | 22 |
| 42 | A2 | 35 | 17 | 15 | 42 | N2 | 53 | 8 | 7 |
| 43 | A2 | 56 | 18 | 17 | 43 | N2 | 56 | 27 | 22 |
| 44 | A2 | 42 | 31 | 28 | 44 | N2 | 78 | 18 | 16 |
| 45 | A2 | 67 | 32 | 19 | 45 | N2 | 17 | 5 | 5 |
| 46 | A2 | 50 | 17 | 8 | 46 | N2 | 70 | 19 | 15 |
| 47 | A2 | 35 | 16 | 6 | 47 | N2 | 73 | 30 | 18 |
| 48 | A2 | 45 | 7 | 5 | 48 | N2 | 55 | 22 | 18 |
| 49 | A2 | 36 | 1 | 1 | 49 | N2 | 55 | 11 | 10 |
| 50 | A2 | 48 | 11 | 11 | 50 | N2 | 55 | 7 | 4 |
| 51 | A2 | 77 | 41 | 33 | 51 | N2 | 60 | 34 | 32 |
| 52 | A2 | 58 | 27 | 18 | 52 | N2 | 56 | 37 | 33 |
| 53 | A2 | 63 | 16 | 14 | 53 | N2 | 68 | 12 | 10 |
| 54 | A2 | 90 | 24 | 23 | 54 | N2 | 53 | 15 | 11 |
| 55 | A2 | 72.5 | 27 | 25 | 55 | N2 | 59.5 | 10 | 8 |
| 56 | A2 | 62 | 19 | 18 | 56 | N3 | 26 | 10 | 8 |
| 57 | A2 | 53 | 13 | 13 | 57 | N3 | 59 | 18 | 17 |
| 58 | A2 | 68 | 18 | 18 | 58 | N3 | 23.5 | 8 | 7 |
| 59 | A2 | 31 | 24 | 19 | 59 | N3 | 51 | 20 | 18 |
| 60 | A2 | 41 | 16 | 15 | 60 | N3 | 9.4 | 5 | 4 |
| 61 | A2 | 48 | 12 | 7 | 61 | N3 | 59 | 29 | 27 |
| 62 | A2 | 44.5 | 18 | 17 | 62 | N3 | 39.5 | 1 | 1 |
| 63 | A2 | 52 | 16 | 16 | 63 | N3 | 30 | 26 | 22 |
| 64 | A2 | 35 | 21 | 18 | 64 | N3 | 16 | 5 | 4 |
| 65 | A2 | 47 | 8 | 5 | 65 | N3 | 35 | 21 | 18 |
| 66 | A3 | 41 | 7 | 4 | 66 | N3 | 27 | 15 | 11 |
| 67 | A3 | 57.4 | 18 | 17 | 67 | N3 | 31 | 23 | 22 |
| 68 | A3 | 79 | 4 | 4 | 68 | N3 | 27.5 | 18 | 17 |
| 69 | A3 | 70 | 8 | 8 | 69 | N3 | 36 | 27 | 26 |
| 70 | A3 | 50 | 4 | 4 | 70 | N3 | 27 | 13 | 11 |
| 71 | A3 | 57 | 7 | 3 | 71 | N3 | 50 | 41 | 35 |
| 72 | A3 | 56 | 6 | 6 | 72 | N3 | 21 | 8 | 5 |
| 73 | A3 | 48 | 19 | 18 | 73 | N3 | 36 | 7 | 3 |
| 74 | A3 | 70 | 21 | 19 | 74 | N3 | 60 | 50 | 47 |
| 75 | A3 | 49 | 22 | 18 | 75 | N3 | 46 | 25 | 13 |
| 76 | A3 | 54 | 18 | 4 | 76 | N3 | 27 | 10 | 6 |
| 77 | A3 | 64 | 4 | 4 | 77 | N3 | 31 | 13 | 7 |
| 78 | A3 | 80 | 12 | 11 | 78 | N3 | 44 | 13 | 6 |
| 79 | A3 | 73 | 15 | 15 | 79 | N3 | 59 | 44 | 40 |
| 80 | A3 | 45 | 17 | 16 | 80 | N3 | 27 | 10 | 7 |
| 81 | A3 | 48 | 4 | 3 | 81 | N3 | 4.4 | 5 | 3 |
| 82 | A3 | 47 | 15 | 6 | 82 | N3 | 48 | 15 | 5 |
| 83 | A3 | 55 | 10 | 9 | 83 | N3 | 16.5 | 19 | 5 |
| 84 | A3 | 45 | 12 | 10 | 84 | N4 | 89 | 49 | 44 |
| 85 | A3 | 60 | 10 | 8 | 85 | N4 | 79 | 41 | 40 |
| 86 | A3 | 40 | 21 | 19 | 86 | N4 | 77.5 | 40 | 30 |
| 87 | A3 | 50 | 16 | 14 | 87 | N4 | 89.5 | 52 | 46 |
| 88 | A4 | 27.6 | 3 | 3 | 88 | N4 | 76 | 42 | 38 |
| 89 | A4 | 41.4 | 8 | 7 | 89 | N4 | 30 | 6 | 2 |
| 90 | A4 | 50 | 17 | 16 | 90 | N4 | 59.5 | 22 | 20 |
| 91 | A4 | 60.3 | 11 | 10 | 91 | N4 | 48 | 15 | 11 |
| 92 | A4 | 48.5 | 9 | 8 | 92 | N4 | 83 | 38 | 20 |
| 93 | A4 | 40 | 9 | 7 | 93 | N4 | 50 | 17 | 10 |
| 94 | A4 | 71 | 15 | 14 | 94 | N4 | 76 | 25 | 17 |
| 95 | A4 | 40 | 9 | 7 | 95 | N4 | 95 | 30 | 16 |
| 96 | A4 | 37 | 1 | 1 | 96 | N4 | 30 | 26 | 20 |
| 97 | A4 | 36.5 | 11 | 9 | 97 | N4 | 16 | 5 | 1 |
| 98 | A4 | 44 | 6 | 6 | 98 | N4 | 35 | 21 | 18 |
| 99 | A4 | 30 | 3 | 0 | 99 | N4 | 27 | 15 | 11 |
| 100 | A4 | 80 | 51 | 50 | 100 | N4 | 31 | 23 | 20 |
| 101 | A4 | 57 | 14 | 12 | 101 | N4 | 27.5 | 18 | 15 |
| 102 | A4 | 30.1 | 1 | 0 | 102 | N4 | 36 | 27 | 22 |
| 103 | A4 | 60 | 27 | 26 | 103 | N4 | 27 | 13 | 10 |
| 104 | A4 | 35 | 10 | 9 | 104 | N4 | 50 | 41 | 33 |
| 105 | A4 | 60 | 7 | 3 | 105 | N4 | 21 | 8 | 3 |
| 106 | A4 | 45 | 11 | 11 | 106 | N4 | 36 | 7 | 5 |
| 107 | A4 | 46 | 11 | 10 | 107 | N4 | 60 | 50 | 46 |
| 108 | A4 | 34 | 12 | 8 | 108 | N4 | 46 | 25 | 22 |
| 109 | A4 | 20 | 1 | 0 | 109 | N4 | 27 | 10 | 10 |
| 110 | A4 | 36 | 12 | 10 | 110 | N4 | 31 | 13 | 11 |
| 111 | A4 | 56 | 19 | 17 | 111 | N4 | 59 | 44 | 38 |
| 112 | A4 | 36 | 2 | 0 | 112 | N4 | 44 | 13 | 11 |
| 113 | A4 | 40 | 10 | 8 | 113 | N4 | 27 | 10 | 8 |
| 114 | A4 | 30 | 1 | 0 | 114 | N4 | 48 | 15 | 11 |
| 115 | A4 | 31 | 1 | 0 | 115 | N4 | 16.5 | 19 | 17 |
| 116 | A4 | 40 | 3 | 3 |  |  |  |  |  |
| 117 | A4 | 46 | 6 | 5 |  |  |  |  |  |
| 118 | A4 | 29.5 | 1 | 0 |  |  |  |  |  |
| 119 | A4 | 39 | 12 | 11 |  |  |  |  |  |
| 120 | A4 | 27.3 | 2 | 1 |  |  |  |  |  |
| 121 | A4 | 35 | 10 | 8 |  |  |  |  |  |
| 122 | A4 | 33 | 1 | 1 |  |  |  |  |  |
| 123 | A4 | 47 | 3 | 1 |  |  |  |  |  |
| 124 | A4 | 37 | 2 | 1 |  |  |  |  |  |
| 125 | A4 | 39 | 2 | 1 |  |  |  |  |  |
|  |  | 2012 | | | | | | | |
| 1 | A1 | 97 | 27 | 27 | 1 | N1 | 68 | 23 | 18 |
| 2 | A1 | 80 | 20 | 20 | 2 | N1 | 70 | 23 | 20 |
| 3 | A1 | 113 | 18 | 18 | 3 | N1 | 73 | 18 | 17 |
| 4 | A1 | 48 | 6 | 6 | 4 | N1 | 73 | 17 | 15 |
| 5 | A1 | 86 | 24 | 22 | 5 | N1 | 75 | 21 | 21 |
| 6 | A1 | 107 | 29 | 27 | 6 | N1 | 74 | 24 | 20 |
| 7 | A1 | 72 | 33 | 30 | 7 | N1 | 71 | 15 | 11 |
| 8 | A1 | 92 | 25 | 25 | 8 | N1 | 80 | 16 | 15 |
| 9 | A1 | 30 | 15 | 14 | 9 | N1 | 75 | 17 | 15 |
| 10 | A1 | 30 | 23 | 22 | 10 | N1 | 85 | 31 | 30 |
| 11 | A1 | 129 | 24 | 24 | 11 | N1 | 44 | 14 | 11 |
| 12 | A1 | 98 | 19 | 19 | 12 | N1 | 54 | 23 | 19 |
| 13 | A1 | 103 | 26 | 27 | 13 | N1 | 42 | 9 | 8 |
| 14 | A1 | 111 | 19 | 19 | 14 | N1 | 71 | 33 | 17 |
| 15 | A1 | 110 | 15 | 15 | 15 | N1 | 45 | 17 | 15 |
| 16 | A1 | 116 | 24 | 22 | 16 | N2 | 72 | 52 | 45 |
| 17 | A1 | 94 | 12 | 11 | 17 | N2 | 60 | 14 | 11 |
| 18 | A1 | 78 | 12 | 11 | 18 | N2 | 87 | 17 | 13 |
| 19 | A1 | 70 | 7 | 7 | 19 | N2 | 69 | 13 | 11 |
| 20 | A1 | 97 | 12 | 12 | 20 | N2 | 78 | 35 | 30 |
| 21 | A1 | 84 | 10 | 10 | 21 | N2 | 90 | 35 | 23 |
| 22 | A1 | 108 | 29 | 28 | 22 | N2 | 80 | 29 | 20 |
| 23 | A1 | 89 | 27 | 27 | 23 | N2 | 66 | 14 | 11 |
| 24 | A1 | 73 | 23 | 23 | 24 | N2 | 65.5 | 28 | 23 |
| 25 | A1 | 93 | 21 | 21 | 25 | N2 | 56 | 7 | 2 |
| 26 | A1 | 98 | 18 | 16 | 26 | N2 | 42 | 5 | 2 |
| 27 | A1 | 83 | 7 | 4 | 27 | N2 | 86 | 22 | 15 |
| 28 | A1 | 95 | 14 | 11 | 28 | N2 | 34 | 9 | 4 |
| 29 | A1 | 98 | 6 | 5 | 29 | N2 | 34.5 | 6 | 5 |
| 30 | A1 | 100 | 26 | 24 | 30 | N2 | 36.5 | 8 | 3 |
| 31 | A1 | 65 | 7 | 7 | 31 | N2 | 50 | 14 | 11 |
| 32 | A1 | 58 | 8 | 8 | 32 | N2 | 54 | 25 | 20 |
| 33 | A1 | 93 | 28 | 27 | 33 | N2 | 73 | 23 | 16 |
| 34 | A1 | 127 | 37 | 36 | 34 | N2 | 79 | 24 | 24 |
| 35 | A1 | 106 | 27 | 27 | 35 | N2 | 60 | 12 | 11 |
| 36 | A1 | 80 | 10 | 10 | 36 | N2 | 56 | 14 | 13 |
| 37 | A1 | 86 | 19 | 18 | 37 | N2 | 51 | 8 | 5 |
| 38 | A1 | 47 | 8 | 8 | 38 | N2 | 63 | 21 | 18 |
| 39 | A1 | 86 | 19 | 19 | 39 | N2 | 49 | 12 | 10 |
| 40 | A1 | 88 | 20 | 20 | 40 | N3 | 44 | 10 | 6 |
| 41 | A1 | 109 | 33 | 33 | 41 | N3 | 35 | 12 | 10 |
| 42 | A1 | 70 | 11 | 11 | 42 | N3 | 68 | 48 | 36 |
| 43 | A1 | 72 | 11 | 11 | 43 | N3 | 70 | 42 | 33 |
| 44 | A2 | 83 | 47 | 46 | 44 | N3 | 73 | 30 | 20 |
| 45 | A2 | 50 | 4 | 4 | 45 | N3 | 73 | 38 | 34 |
| 46 | A2 | 70 | 25 | 22 | 46 | N3 | 75 | 39 | 30 |
| 47 | A2 | 58 | 16 | 15 | 47 | N3 | 74 | 35 | 22 |
| 48 | A2 | 40 | 1 | 1 | 48 | N3 | 71 | 36 | 20 |
| 49 | A2 | 26 | 5 | 5 | 49 | N3 | 54 | 23 | 13 |
| 50 | A2 | 48 | 2 | 2 | 50 | N3 | 80 | 43 | 30 |
| 51 | A2 | 50 | 28 | 28 | 51 | N3 | 75 | 32 | 16 |
| 52 | A2 | 55 | 20 | 20 | 52 | N3 | 85 | 33 | 19 |
| 53 | A2 | 58 | 11 | 11 | 53 | N3 | 44 | 14 | 11 |
| 54 | A2 | 70 | 20 | 20 | 54 | N3 | 42 | 9 | 5 |
| 55 | A2 | 26 | 1 | 1 | 55 | N3 | 71 | 33 | 20 |
| 56 | A2 | 43 | 4 | 4 | 56 | N3 | 54 | 8 | 3 |
| 57 | A2 | 48 | 14 | 14 | 57 | N3 | 45 | 17 | 14 |
| 58 | A2 | 36 | 7 | 7 | 58 | N3 | 26 | 10 | 5 |
| 59 | A2 | 45 | 12 | 11 | 59 | N3 | 59 | 18 | 6 |
| 60 | A2 | 30 | 3 | 3 | 60 | N3 | 23.5 | 8 | 4 |
| 61 | A2 | 52 | 10 | 10 | 61 | N3 | 31 | 20 | 7 |
| 62 | A2 | 71 | 24 | 24 | 62 | N3 | 9.4 | 5 | 5 |
| 63 | A2 | 85 | 36 | 36 | 63 | N3 | 59 | 29 | 9 |
| 64 | A2 | 73 | 33 | 30 | 64 | N3 | 39.5 | 1 | 9 |
| 65 | A2 | 65 | 21 | 20 | 65 | N4 | 30 | 26 | 20 |
| 66 | A2 | 59 | 16 | 16 | 66 | N4 | 16 | 5 | 4 |
| 67 | A2 | 99 | 62 | 58 | 67 | N4 | 35 | 21 | 11 |
| 68 | A2 | 80 | 35 | 34 | 68 | N4 | 27 | 15 | 11 |
| 69 | A2 | 62 | 31 | 31 | 69 | N4 | 31 | 23 | 15 |
| 70 | A2 | 90 | 23 | 23 | 70 | N4 | 27.5 | 18 | 14 |
| 71 | A2 | 62 | 18 | 18 | 71 | N4 | 36 | 27 | 13 |
| 72 | A2 | 70 | 28 | 28 | 72 | N4 | 27 | 13 | 12 |
| 73 | A2 | 88 | 38 | 37 | 73 | N4 | 50 | 41 | 35 |
| 74 | A2 | 70 | 26 | 25 | 74 | N4 | 21 | 8 | 5 |
| 75 | A2 | 69 | 23 | 23 | 75 | N4 | 36 | 7 | 3 |
| 76 | A2 | 91 | 48 | 48 | 76 | N4 | 60 | 50 | 40 |
| 77 | A2 | 68 | 11 | 11 | 77 | N4 | 46 | 25 | 20 |
| 78 | A2 | 50 | 7 | 7 | 78 | N4 | 27 | 10 | 6 |
| 79 | A2 | 49 | 4 | 4 | 79 | N4 | 31 | 13 | 7 |
| 80 | A2 | 74 | 33 | 30 | 80 | N4 | 44 | 13 | 6 |
| 81 | A2 | 92 | 52 | 48 | 81 | N4 | 59 | 44 | 30 |
| 82 | A2 | 66 | 11 | 10 | 82 | N4 | 27 | 10 | 7 |
| 83 | A2 | 73 | 12 | 12 | 83 | N4 | 48 | 15 | 5 |
| 84 | A2 | 94 | 19 | 19 | 84 | N4 | 16.5 | 19 | 15 |
| 85 | A2 | 107 | 27 | 27 | 85 | N4 | 89 | 49 | 45 |
| 86 | A2 | 81 | 18 | 18 | 86 | N4 | 74 | 41 | 42 |
| 87 | A2 | 48 | 7 | 7 | 87 | N4 | 77.5 | 40 | 39 |
| 88 | A2 | 71 | 27 | 27 | 88 | N4 | 89.5 | 52 | 49 |
| 89 | A3 | 45 | 4 | 3 | 89 | N4 | 7.6 | 42 | 40 |
| 90 | A3 | 56 | 2 | 2 | 90 | N4 | 30 | 6 | 5 |
| 91 | A3 | 57 | 1 | 1 | 91 | N4 | 59.5 | 22 | 22 |
| 92 | A3 | 55 | 2 | 2 | 92 | N4 | 48 | 15 | 11 |
| 93 | A3 | 45 | 2 | 2 | 93 | N4 | 83 | 38 | 35 |
| 94 | A3 | 42 | 5 | 4 | 94 | N4 | 50 | 27 | 24 |
| 95 | A3 | 21 | 5 | 4 | 95 | N4 | 76 | 25 | 22 |
| 96 | A3 | 70 | 6 | 0 | 96 | N4 | 95 | 30 | 19 |
| 97 | A3 | 88 | 7 | 5 |  |  |  |  |  |
| 98 | A3 | 90 | 25 | 23 |  |  |  |  |  |
| 99 | A3 | 67 | 10 | 7 |  |  |  |  |  |
| 100 | A3 | 45 | 4 | 3 |  |  |  |  |  |
| 101 | A3 | 47 | 6 | 4 |  |  |  |  |  |
| 102 | A4 | 25 | 8 | 6 |  |  |  |  |  |
| 103 | A4 | 25 | 8 | 8 |  |  |  |  |  |
| 104 | A4 | 45 | 19 | 17 |  |  |  |  |  |
| 105 | A4 | 47 | 13 | 10 |  |  |  |  |  |
| 106 | A4 | 78 | 12 | 9 |  |  |  |  |  |
| 107 | A4 | 43 | 10 | 8 |  |  |  |  |  |
| 108 | A4 | 69 | 17 | 14 |  |  |  |  |  |
| 109 | A4 | 50 | 9 | 7 |  |  |  |  |  |
| 110 | A4 | 43 | 1 | 1 |  |  |  |  |  |
| 111 | A4 | 23 | 14 | 11 |  |  |  |  |  |
| 112 | A4 | 55 | 10 | 7 |  |  |  |  |  |
| 113 | A4 | 35 | 3 | 0 |  |  |  |  |  |
| 114 | A4 | 78 | 51 | 50 |  |  |  |  |  |
| 115 | A4 | 38 | 14 | 12 |  |  |  |  |  |
| 116 | A4 | 38 | 1 | 0 |  |  |  |  |  |
| 117 | A4 | 78 | 27 | 26 |  |  |  |  |  |
| 118 | A4 | 40 | 12 | 12 |  |  |  |  |  |
| 119 | A4 | 55 | 6 | 6 |  |  |  |  |  |
| 120 | A4 | 47 | 11 | 11 |  |  |  |  |  |
| 121 | A4 | 58 | 11 | 10 |  |  |  |  |  |
| 122 | A4 | 35 | 12 | 8 |  |  |  |  |  |
| 123 | A4 | 34 | 1 | 0 |  |  |  |  |  |
| 124 | A4 | 37 | 12 | 11 |  |  |  |  |  |
| 125 | A4 | 57 | 19 | 16 |  |  |  |  |  |
| 126 | A4 | 25 | 2 | 0 |  |  |  |  |  |
| 127 | A4 | 36 | 10 | 8 |  |  |  |  |  |
| 128 | A4 | 26 | 1 | 0 |  |  |  |  |  |
| 129 | A4 | 19 | 1 | 0 |  |  |  |  |  |
| 130 | A4 | 33 | 6 | 5 |  |  |  |  |  |
| 131 | A4 | 23 | 6 | 0 |  |  |  |  |  |
| 132 | A4 | 28 | 1 | 0 |  |  |  |  |  |
| 133 | A4 | 40 | 12 | 10 |  |  |  |  |  |
| 134 | A4 | 30 | 3 | 1 |  |  |  |  |  |
| 135 | A4 | 39 | 10 | 8 |  |  |  |  |  |
| 136 | A4 | 26 | 3 | 1 |  |  |  |  |  |
| 137 | A4 | 35 | 3 | 1 |  |  |  |  |  |
| 138 | A4 | 17 | 5 | 4 |  |  |  |  |  |
| 139 | A4 | 26.5 | 2 | 1 |  |  |  |  |  |
| 140 | A4 | 28 | 1 | 0 |  |  |  |  |  |
| 141 | A4 | 70 | 12 | 10 |  |  |  |  |  |
| 142 | A4 | 39 | 3 | 1 |  |  |  |  |  |
| 143 | A4 | 41 | 9 | 8 |  |  |  |  |  |
| 144 | A4 | 28 | 4 | 1 |  |  |  |  |  |
| 145 | A4 | 55 | 0 | 1 |  |  |  |  |  |
| 146 | A4 | 27 | 4 | 4 |  |  |  |  |  |
| 147 | A4 | 26.5 | 2 | 1 |  |  |  |  |  |
