# Supplementary material for: Pollinator diversity and reproductive success of Epipactis helleborine (L.) Crantz (Orchidaceae) in anthropogenic and natural habitats
Source: PeerJ. 2017 Apr 18;5:e3159. doi: 10.7717/peerj.3159 (PMC5398293; doi:10.7717/peerj.3159)
Supplement: Table S3 — Explanations: population (Pop), density (D), number of flowers (NF), number of fruit (capsules) (NFR), reproductive success (RS). [file peerj-05-3159-s003.docx]

Supplementary Table 3. Raw data - average of biometric traits of *E. helleborine* in the studied populations. Explanations: population (Pop), density (D), number of flowers (NF), number of fruit (capsules) (NFR), reproductive success (RS).

| Pop | D | NF | NFR | RS |
| --- | --- | --- | --- | --- |
| 2011 | | | | |
| A1 | 0.28 | 20 | 19 | 95 |
| A2 | 0.93 | 19 | 16 | 84.2 |
| A3 | 0.9 | 12 | 10 | 83.3 |
| A4 | 0.4 | 9 | 7 | 77.8 |
| Average | 0.6275 | 15 | 13 | 85.075 |
| A1 | 0.38 | 19 | 18 | 94.7 |
| A2 | 0.77 | 20 | 20 | 100 |
| A3 | 2.39 | 6 | 5 | 83.3 |
| A4 | 0.33 | 9 | 7 | 77.8 |
| Average | 0.9675 | 14 | 12 | 88.95 |
|  | D | NF | NFR | RS |
| 2012 | | | | |
| N1 | 0.83 | 18 | 8 | 44.4 |
| N2 | 0.77 | 18 | 13 | 72.2 |
| N3 | 0.21 | 18 | 14 | 77.8 |
| N4 | 0.88 | 24 | 20 | 83.3 |
| Average | 0.6725 | 20 | 14 | 69.425 |
| N1 | 1.24 | 20 | 17 | 85 |
| N2 | 0.97 | 19 | 14 | 73.7 |
| N3 | 0.22 | 24 | 15 | 62.5 |
| N4 | 0.81 | 25 | 20 | 80 |
| Average | 0.81 | 22 | 17 | 75.3 |
