# Supplementary material for: Pollinator diversity and reproductive success of Epipactis helleborine (L.) Crantz (Orchidaceae) in anthropogenic and natural habitats
Source: PeerJ. 2017 Apr 18;5:e3159. doi: 10.7717/peerj.3159 (PMC5398293; doi:10.7717/peerj.3159)
Supplement: Table S4 [file peerj-05-3159-s004.docx]

Supplementary Table S4. Raw data - average of live and dead seeds [%] in autogamy and natural pollinations of *E. helleborine* in the studied populations, calculated from 10 shoots from each population.

|  | 1 | 2 | 3 | 4 | 5 | 6 | 7 | 8 | 9 | 10 |
| --- | --- | --- | --- | --- | --- | --- | --- | --- | --- | --- |
| **Natural pollination** | live | | | | | | | | | |
| Anthropogenic | 50.5 | 54 | 48 | 47 | 49 | 53 | 52 | 50 | 48 | 46 |
| Natural | 55 | 53 | 50 | 50 | 48 | 55 | 52 | 55 | 48 | 46 |
|  | dead | | | | | | | | | |
| Anthropogenic | 49.5 | 45 | 51 | 52 | 51 | 52 | 47 | 50 | 52 | 54 |
| Natural | 45 | 47 | 50 | 50 | 52 | 45 | 47 | 45 | 52 | 53.5 |
| **Autogamy** |  |  |  |  |  |  |  |  |  |  |
|  | live | | | | | | | | | |
| Anthropogenic | 25.5 | 23 | 24 | 25 | 25 | 26 | 27 | 30 | 20 | 20 |
| Natural | 30 | 20 | 28 | 25 | 25 | 32 | 30 | 34 | 36 | 35 |
|  | dead | | | | | | | | | |
| Anthropogenic | 75 | 77 | 75.5 | 75 | 75 | 73.5 | 73 | 70 | 80 | 80 |
| Natural | 70 | 80 | 71.5 | 75 | 75 | 68 | 70 | 66 | 64 | 65 |
